# Evaluation of Antifungal Activity and Mechanism of Action of Citral against *Candida albicans*

**DOI:** 10.1155/2014/378280

**Published:** 2014-08-28

**Authors:** Maria Clerya Alvino Leite, André Parente de Brito Bezerra, Janiere Pereira de Sousa, Felipe Queiroga Sarmento Guerra, Edeltrudes de Oliveira Lima

**Affiliations:** ^1^Federal Institute of Education, Science, and Technology of Paraíba (IFPB), 58780-000 Itaporanga, PB, Brazil; ^2^Mycology Laboratory, Department of Pharmaceutical Sciences, Federal University of Paraíba, 58051-970 João Pessoa, PB, Brazil

## Abstract

*Candida albicans* is a yeast that commensally inhabits the human body and can cause opportunistic or pathogenic infections. *Objective*. To investigate the antifungal activity of citral against *C. albicans*. *Methodology*. The minimum inhibitory concentration (MIC) and the minimum fungicidal concentration (MFC) were determined by the broth microdilution techniques. We also investigated possible citral action on cell walls (0.8 M sorbitol), cell membranes (citral to ergosterol binding), the time-kill curve, and biological activity on the yeast's morphology. *Results*. The MIC and MFC of citral were, respectively, 64 *µ*g/mL and 256 *µ*g/mL. Involvement with the cell wall and ergosterol binding were excluded as possible mechanisms of action. In the morphological interference assay, it was observed that the product inhibited pseudohyphae and chlamydoconidia formation. The MIC and the MFC of citral required only 4 hours of exposure to effectively kill 99.9% of the inoculum. *Conclusion*. Citral showed *in vitro* antifungal potential against strains of *C. albicans*. Citral's mechanism of action does not involve the cell wall or ergosterol, and further study is needed to completely describe its effects before being used in the future as a component of new antifungals.

## 1. Introduction

The prevalence of* Candida albicans* infections is increasing at an alarming rate, and this is especially true for immunocompromised individuals, such as AIDS patients, transplant patients, and neonates [1, 2].* C. albicans* is an opportunist pathogen that lives commensally within the human body; it is the leading cause of human fungal infections.* C. albicans* infection usually develops as a consequence of host immune response alterations [3].

The increased incidence of* Candida* infections can be attributed to a variety of factors, either exogenous or (especially) endogenous. Over 100 species of* Candida* are known;* C. albicans* is the main representative. The frequency of distribution for* Candida *spp. varies in accordance with geographical location. Among the* Candida* species, it is* C. albicans* that is involved in bloodstream infections for 44% of Latin American and 62% of European cases [4].

Conventional fungal infection treatments are unsatisfactory. Therefore, it has become essential to develop new drugs and alternative therapies (including natural products) for the treatment of* C. albicans* infections. Plants and their derivatives are known to be important in pharmacological research due to their great potential as a source for a variety of biologically active ingredients used in drug development. Amongst these products we find the terpenes, a class of natural substances of vegetable origin formed by combining five carbons called isoprene (C_5_H_8_). Terpenes can be classified according to their number of isoprene units: monoterpenes (C_10_), the most representative molecules, and sesquiterpenes (C_15_), but there are also hemiterpenes (C_5_), diterpenes (C_20_), triterpenes (C_30_), and tetraterpenes (C_40_) [5].

Citral (3,7-dimethyl-2-6-octadienal) is the name given to a mixture of two geometric isomers: geranial (*trans*-citral, citral A) and neral (*cis*-citral, citral B), which are acyclic α, *β*-unsaturated monoterpene aldehydes that occur naturally in many essential citrus fruit oils and in other herbs or spices [6–10]. The citral aroma is stronger and sweeter than that of lemon. Geranial has a strong lemon odor while neral has a sweeter, yet less intense lemon odor. Due to its characteristic lemon aroma, citral has become a flavoring substance of great importance, a heavily used raw material for the pharmaceutical, food, perfume, and cosmetics industries [11, 12].

The antifungal activity exerted by citral against molds and yeasts has already been demonstrated in varied conditions [10, 13–20]. Recently, it was demonstrated that citral has the ability to destroy the integrity of the cell membrane, releasing the cellular components of* Geotrichum citri-aurantii* [21] and dramatically inhibiting the mycelial growth of* Penicillium italicum* through a mechanism of cell membrane damage, compromising its integrity and permeability [22]. Da silva et al. [23] have demonstrated that citral exhibits significant* in vitro* activity against* C. glabrata, C. krusei, C. parapsilosis, and C. tropicalis*, and especially against species of* C. albicans*. Zore et al. [24] demonstrated the anti-*Candida* activity of six terpenoids; all showed excellent activity against* C. albicans *isolates, being the most effective linalool and citral.

Given the above, the aim of this study was to determine the minimum inhibitory concentration (MIC) and the minimum fungicidal concentration (MFC) and to investigate the action mechanism of citral against* C. albicans* in its growth kinetics, micromorphology, cell wall formation, and ergosterol interactions.

## 2. Materials and Methods

### 2.1. Phytoconstituent, Antifungal Standard, and Substances

The following substances used in this work were obtained commercially: citral (purity 95%), amphotericin B, ergosterol, and sorbitol (all from Sigma-Aldrich, São Paulo, SP, Brazil). Furthermore, dimethylsulfoxide (DMSO) and Tween-80 were, respectively, purchased from Labsynth Products for Laboratories Ltd. (Diadema, SP, Brazil) and Vetec Fine Chemicals Ltd. (Duque de Caxias, RJ, Brazil).

### 2.2. Culture Media

To test the biological activity of the products, Sabouraud dextrose broth (SDB) and Sabouraud dextrose agar (SDA) were purchased from Difco Laboratories (Detroit, MI, USA) and agar-cornmeal from HiMédia Laboratories (Mumbai, India), and RPMI-1640-L-glutamine (without sodium bicarbonate) (Sigma-Aldrich, São Paulo, SP, Brazil) culture media were used. They were prepared and used according to the manufacturers' instructions.

### 2.3. Fungal Strains

The assays were performed with 10 strains of* C. albicans*: LM-14, LM-17, LM-70, and LM-520 (isolated from blood cultures), LM-11, LM-15, LM-94, and LM-410 (lung secretion), and two standard* C. albicans* strains: ATCC 76485 and ICB-12. All strains belong to the collection of the Mycology Laboratory, Department of Pharmaceutical Sciences, Federal University of Paraíba (LM, DCF, UFPB). These strains were maintained in SDA at 35°C and 4°C until used in tests.

### 2.4. Inoculum Preparation

The suspensions were prepared from recent* C. albicans* cultures, plated on SDA, and incubated at 35°C for 24–48 h. After incubation, we transferred roughly 4-5 yeast colonies (with a sterile loop) to test tubes containing 5 mL of saline solution 0.9% (Farmax-Distributor Ltd., Amaral, Divinópolis, MG, Brazil). The resulting suspensions were stirred for 15 seconds with the aid of a Vortex apparatus (Fanem Ltd., Guarulhos, SP, Brazil).

The turbidity of the final inoculum was standardized using a barium sulfate suspension (tube 0.5 on the McFarland scale). The final concentration obtained was about 1–5 × 10^6^ colony forming units per milliliter (CFU/mL). The final concentration confirmation was done by counting the microorganisms in a Neubauer chamber [25–27].

### 2.5. Determination of Minimum Inhibitory Concentration (MIC) and Minimum Fungicidal Concentration (MFC)

The determination of the products' MIC on the ten strains used in the biological assays was determined by the broth microdilution method [25–27]. One hundred milliliters (100 *μ*L) of liquid medium RPMI-1640 was transferred into the wells of a 96-well microdilution plate with a “U” shaped bottom (Alamar, Diadema, SP, Brazil). Then, 100 *μ*L of citral emulsion was inoculated in the first horizontal row of the plate wells. Doubled serial dilutions, where a 100 *μ*L aliquot removed from the most concentrated well went to the next well, yielded concentrations of 1024-1 *μ*g/mL. Finally, 10 *μ*L of* C. albicans* inoculum suspension was added to each well of the plate, where each column represented a yeast strain. In parallel, controls were made for yeast viability and for susceptibility with the standard antifungal amphotericin B. The plates were incubated at 35°C for 24–48 h. After the appropriate incubation time, the presence (or absence) of growth was observed visually. The formation of cell clusters or “buttons” in the plate wells was considered. The MIC was defined as the lowest citral concentration that produced visible inhibition of yeast growth.

The antimicrobial activity of the products was interpreted (considered active or not), according to the criteria proposed by Morales et al. [28]: strong/good activity (MIC: <100 *μ*g/mL); moderate activity (MIC: 100–500 *μ*g/mL); weak activity (MIC: 500–1000 *μ*g/mL); and inactive product/no antimicrobial effect (MIC: >1000 *μ*g/mL).

To determine the MFC, we subcultured 1 *μ*L aliquots of MIC, MIC × 2, and MIC × 4 of the citral products, amphotericin B, and the control yeast growth onto Petri dishes containing SDA. After 24–48 hours of incubation at 35°C, a reading was made to evaluate the MFC, as based on the growth of the controls. The MFC was defined as the lowest product concentration that inhibited growth of the yeast or permitted less than three CFUs to occur, resulting thus in 99.9% fungicidal activity [29, 30].

Biological activity assays were performed in duplicate, and the results were expressed as the arithmetic mean of the MIC and MFC.

### 2.6. Time-Kill Curve

The study of citral and amphotericin B interference on the death curve times for* C. albicans* was conducted with methodology described by Klepser et al. [31], with some modifications, using the method of counting viable cells. To determine the kinetics of fungal death, two strains were selected: a standard strain ATCC 76485 and a clinical strain LM-70, in accordance with the MIC and MFC results. In this test, the behavior of selected yeast strains was observed for 24 h.

A series of sterile test tubes was prepared and we added 4.5 mL of RPMI 1640 medium containing various product concentrations: MIC/2, MIC, and MIC × 2. We then added 0.5 mL of the yeast suspension and the assay was incubated at 35°C for 24 h. Ten-microliter (10 *μ*L) aliquots were removed with a sterile calibrated loop at predetermined time intervals (0 h, 2 h, 4 h, 6 h, and 24 h) and uniformly seeded on the surface of SDA culture medium. The assay was incubated at 35°C for 24–48 h, and at the elapsed incubation time, the count was done (CFU/mL). Controls for yeast growth and the antifungal standard were also performed. The experiment was performed in duplicate.

After the incubation period, the number of viable cells was counted and expressed in CFU/mL. The results were analyzed and represented graphically, a microbial death curve as a function of time. Fungicidal activity of the product-test was considered when there was a reduction in microbial growth of ≥3 log_10_ in CFU/mL, resulting in about 99.9% reduction in CFU/mL relative to the initial inoculum. Fungistatic activity was considered as reduction in growth lower than 99.9% or <3 log_10_ in CFU/mL from the initial inoculum [31].

### 2.7. Effect of Citral on the Micromorphology of* C. albicans*

To evaluate possible changes in micromorphology caused by citral and amphotericin B against* C. albicans* ATCC 76485 and LM-70, microculture technique for yeast was employed using agar-cornmeal in a moist chamber/Petri dish, in accordance with Dalmau [32]. Initially, 1 mL of liquefied cornmeal agar containing the test product was poured onto a slide. After solidification of the medium, an inoculum of recent cultures of the strains was seeded forming two parallel striations on the cornmeal agar and covered with a sterile coverslip. The filter paper was then soaked in sterile distilled water to maintain moisture in the system. This test system was incubated at 35°C for 24–48 h. The slides were examined by light microscopy at a magnification of 40x to observe the formation (or not) of characteristic structures such as blastoconidia, pseudohyphae, and chlamydoconidia, and images were registered. Microculture controls (including the antifungal standard) were also completed [33–35]. The test was performed in duplicate.

### 2.8. Sorbitol Assay-Effect of Citral on the Cell Wall of* C. albicans*

The assay was performed using medium with and without sorbitol (control) to evaluate possible mechanisms involved in the antifungal activity of the test product on the yeast cell wall. The sorbitol was added to the culture medium in a final concentration of 0.8 M. The assay was performed by microdilution method in 96-well plates in a “U” (Alamar, Diadema, SP, Brazil). The plates were sealed aseptically, incubated at 35°C, and readings were taken at 2 and 7 days. Based on the ability of sorbitol to act as a fungal cell wall osmotic protective agent, the higher MIC values observed in the medium with added sorbitol compared to the standard medium implicated the cell wall as one of the possible cell targets for the product tested [36]. Amphotericin B was used as the control drug. The assay was performed in duplicate and expressed as the geometric mean of the results.

### 2.9. Ergosterol Binding Assay-MIC Value Determination in Presence of Ergosterol

To assess if the product binds to the fungal membrane sterols, this experiment was performed according to the method described by Escalante et al. [37], with some modifications. The ergosterol was prepared at the time of test execution, where it was first pulverized (with the help of a pre-sterilized porcelain mortar and pestle) and dissolved in DMSO (no more than 10% of final volume), and Tween 80 at 1%, in accordance with the desired concentration and volume. The formed emulsion was then homogenized, heated to augment the solubility, and diluted with the liquid culture medium.

The MIC of citral against* C. albicans* was determined by using broth microdilution techniques according to the guidelines of the CLSI for yeasts (M27-A2) [27], in the presence and absence of exogenous ergosterol (Sigma-Aldrich, São Paulo, SP, Brazil) added to the assay medium, in different lines of the same microplate. Briefly, a solution of citral was doubly diluted serially with RPMI-1640 (volume = 100 *μ*L) containing plus ergosterol at a concentration of 400 *μ*g/mL. A volume of 10 *μ*L of yeast suspension (0,5 McFarland) was added to each well. Finally, we realized the same procedure for amphotericin B, whose interaction with membrane ergosterol is already known, which served as a control drug. The plates were sealed and incubated at 35°C. The plates were read after 24 h of incubation and MIC was determined as the lowest concentration of test agent inhibiting the visible growth. This assay was carried out in duplicate and the geometric mean values were calculated. Thus, this binding assay reflected the ability of compound to bind with the ergosterol.

## 3. Results and Discussion

The results for citral's antifungal activity against* C. albicans* strains were determined using the MIC and MFC in broth microdilutions. The MIC of citral was 64 *μ*g/mL, inhibiting the growth of all tested fungal strains. Amphotericin B retained a lesser MIC than the phytoconstituent at 2 *μ*g/mL MIC. The results for the control (Tween 80) showed no fungal growth inhibition; fungal growth in the medium without added drug was detected (sterile control).

The MFC against these microorganisms almost entirely coincided with the MIC (64 *μ*g/mL), except that for the LM-11 strain it was 256 *μ*g/mL. However, the MFC of amphotericin B ranged from 2 to 4 *μ*g/mL, eight strains (with MFC equal to the MIC), and two strains with 2 times the MIC value (4 *μ*g/mL).

In accordance with the above results, the strains ATCC 76485 and LM-70 were selected for further testing. The MIC for citral and for amphotericin B both strains was 64 and 2 *μ*g/mL, respectively.

The antimicrobial activity of citral has been confirmed* in vitro* against* Saccharomyces cerevisiae* [13] and also in fruit salad [15]. This has been attributed to its high concentrations of citral, whose antimicrobial potential is known, and reported by other authors [10, 12, 16, 20, 38]. More recently, studies have shown that citral can also be used as an antiprotozoal drug. Cardoso and Soares [39] have shown that citral effectively blocked the differentiation from epimastigote to trypomastigote (metacyclogenesis) of* Trypanosoma cruzi in vitro*. Citral may be a good candidate drug for new inhibition studies to analyze the process of metacyclogenesis for* T. cruzi *[39].

It has been reported that citral (at 25–200 *μ*g/mL) and lemongrass oil,* Cymbopogon citratus* (at 100 *μ*g/mL), have antifungal activity, inhibiting the mycelial growth of* C. albicans*, which suggests the potential value of this oil to treat oral or vaginal candidiasis. The lemongrass oil is characterized by monoterpene compounds, and citral is the principal component present at levels of approximately 80% [40]. Similarly, De billerbeck et al. [41] demonstrated that citral was responsible for 70–80% of the antifungal activity of* Cymbopogon citratus* essential oil.

In the present study, citral showed activity against* C. albicans* isolates, confirming the results obtained in previous studies [12, 23, 42–45]. However, citral exhibited excellent activity, even more effective than previously reported, where the compound exhibited antifungal activity in concentrations greater than 256 *μ*g/mL against* C. albicans* [24, 42–44].

The product was therefore considered actively antifungal in accordance with the parameters defined by Morales et al. [28].

**Figure 1:**
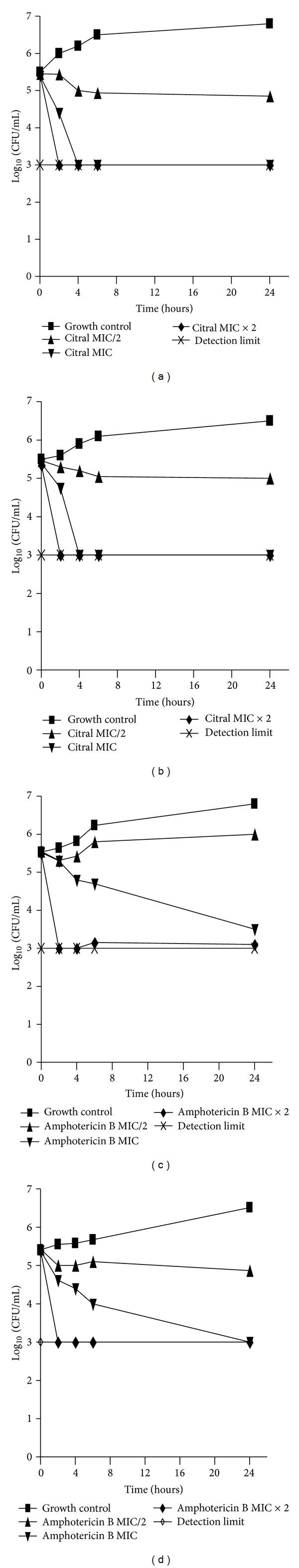
(a) and (c) Time-death curve for* C. albicans* ATCC 76485 when exposed to various concentrations of citral and amphotericin B, respectively. (b) and (d) Time-death curve for* C. albicans* LM-70 when exposed to various concentrations of citral and amphotericin B, respectively.

Yeast growth was analyzed over time while subjected to various concentrations of the test product. Two* C. albicans* strains (ATCC 76485 and LM 70) were subjected to the experimental method for microbial death kinetics (Figure 1). The test realizes a viable cell count, checking whether a drug has fungistatic or fungicidal action, as well as analyzing the microorganism-test product interaction, in order to characterize a dynamic relationship between concentration and activity over time [31].

The graphs show the log_10_ of CFU/mL versus time of exposure in the presence of citral (MIC/2, MIC, and MIC × 2), the standard antifungal, and the control. Analysis of the graph reveals that the citral concentration MIC/2 has fungistatic activity, as there was a reduction smaller than 3 log_10_ CFU/mL of the initial inoculum; this behavior was also seen for the MIC at up to 2 hours of exposure. At 4 hours' exposure time to citral at concentrations of MIC or MIC × 2, we see fungicidal activity (reduction above 3 log_10_ of CFU/mL of initial inoculum). Therefore, it was possible to identify in the death kinetics a transition between fungistatic and fungicidal activity, revealing citral's concentration-dependent fungicidal activity. The results obtained in this study for* C. albicans* in the presence of citral resemble those of Zore et al. [24] also for* C. albicans*, with regard to the decrease of cell viability in the initial 2 hours. However, the authors noted that 2 hours were required to kill 99.9% of the inoculum at a concentration of 640 *μ*g/mL. In the present study, the time required for presentation of fungicidal activity was 2 hours for MIC × 2 and 4 hours for MIC. Essential oil rich in citral has shown microbiocidic activity against* C. albicans*,* Escherichia coli*, and* Staphylococcus aureus*, being able to “instantly” kill (10 min) at a concentration of 1 mg/mL, indicating its broad spectrum of antimicrobial activity in easily achievable concentrations [46].

As shown in Figures 1(c) and 1(d), the time-kill assay, a 100% reduction in* C. albicans* viability was observed with exposure of 24 h for MIC and of 2 h for MIC × 2 with amphotericin B. These results confirm the results obtained by Cantón et al. [47] where the fungicidal activity of amphotericin B against* C. albicans* was very quick (2 to 4 hours in concentrations equal to MIC × 2), with a decrease in the number of CFU per milliliter that was greater than 3 log_10_ units (99.9%). The literature has reported that, among the drugs commonly used to treat fungal infections, amphotericin B and nystatin have concentration-dependent fungicidal effect [31].

**Figure 2:**
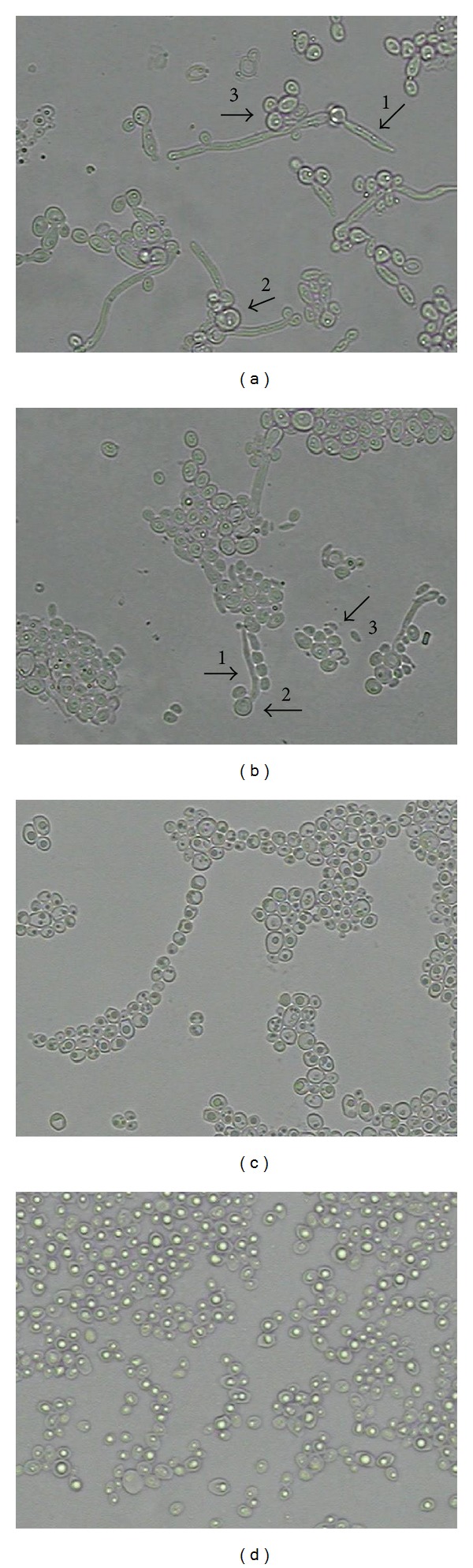
Micromorphology of* Candida albicans* strains in the absence (control) and presence of citral and amphotericin B. (a)* C. albicans* ATCC 76485 and (b)* C. albicans* LM-70 (in the absence of the product) as controls showing the presence of blastoconidia, chlamydoconidia, and pseudohyphae. (c) Under the action of amphotericin B—MIC. (d) Under the action of citral—MIC × 2. (1) Pseudohyphae; (2) chlamydoconidia; and (3) blastoconidia.

The micromorphological evaluations of* C. albicans* ATCC 76485 and LM-70 under an optical microscope (for the control) revealed the presence of structures indicating fungal growth: pseudohyphae, blastoconidia, and chlamydoconidia (Figure 2). In the presence of citral we observed only blastoconidia. Thus, the fungal micromorphological assay indicated that the test product was able to inhibit the formation of pseudohyphae and chlamydoconidia. The same was observed for amphotericin B. A previous study has shown that lemongrass essential oil is rich in citral, and having a concentration of 32.7 *μ*g/mL is highly effective in the vapor phase against* C. albicans*, causing deleterious morphological changes in cellular structures and cell surface alterations [48, 49].

According to Alves et al. [50], morphological changes are associated with microorganism pathogenicity, and it is believed that local environmental factors affect the physiological status of commensal yeast, making them infectious.

The yeast-hyphae morphological transition is considered highly relevant to the virulence of fungal infections. Several studies have reported associations between morphogenesis and virulence for dimorphic fungi that are human pathogens [51–55]. Thus, change in morphology from the yeast form to the filamentous form plays a vital role in the pathogenesis of fungal infections and suggests that associated factors for this conversion process represent promising therapeutic targets [3, 51]. There are reports indicating that mutant* C. albicans* strains incapable of hyphal formation are, generally, nonvirulent in mouse models of experimental disseminated or mucosal candidiasis [56–59]. The results for citral in this work may therefore be of great importance towards the development of some future antifungal agent.

To investigate the action of the product on the fungal cell wall we performed an assay with sorbitol (Table 1), which has an osmoprotectant function. Sorbitol is an osmotic protector used to stabilize fungi protoplasts. Specific fungal cell wall inhibitors share a distinctive characteristic where their antifungal effects are reversed in mediums containing sorbitol [36]. Cells protected with sorbitol can grow in the presence of fungal cell wall inhibitors, whereas growth would be inhibited in the absence of sorbitol. This effect is detected by increases in the MIC value as observed in medium with sorbitol as compared to the MIC value in medium without sorbitol (standard medium) [36, 60]. Osmotic destabilizing agents and disrupting the cell wall lead to rearrangements of the cell wall and allow the fungal cells to survive [27].

In this paper, the MIC values of citral in both experiments, in mediums with and without sorbitol, were identical, suggesting that citral does not act by inhibiting fungal cell wall synthesis, but probably by affecting another target. These results are in agreement with those reported by Lima et al. [44] who have also described the antifungal activity of this volatile compound against* C. albicans*.

According to Harris [61], citral appears to act predominantly on the fungal cell membrane, affecting its structure, blocking its synthesis, and causing cell death, while inhibiting spore germination, proliferation, and cellular respiration. The literature suggests that the antifungal activity of citral is due to its ability to form a charge transfer complex with fungal cell tryptophan, resulting in the death of the fungi [62].

Considering this possible fungal cell membrane interference of citral, the compound was tested to investigate its ability to form complexes with ergosterol (Table 1).

Ergosterol is the major sterol component present in the plasma membrane of fungi and plays the same role in fungal membranes that cholesterol plays in mammalian cell membranes [63]. Thus, these two sterols seem to exhibit qualitatively similar properties.

If the activity of citral is caused by binding to ergosterol, the exogenous ergosterol would prevent the binding to ergosterol in the fungal membranes. Consequently, MIC increase for citral (in the presence of exogenous ergosterol in relation to the control assay) would occur because only increased product concentration in the growth medium might assure interaction with ergosterol in the fungal membranes [37, 64]. Thus, the effect of exogenous ergosterol on citral and amphotericin B MIC was determined. As can be seen, citral displayed no changes in MIC values; the values were identical in medium with and without additional ergosterol. This indicates that the mechanism of action of citral does not involve complexation with ergosterol. However, amphotericin B does complicate with ergosterol, thus showing an increase in its MIC of 64 times (Table 1). These results are consistent with previous studies for* C. albicans*, in which, in the presence of ergosterol, the MIC value of amphotericin B increased 32 times; the same was not observed for the MIC of citral [54]. However, the results obtained recently by Rajput and Karuppayil [65] demonstrate that citral used at the CIM value altered the ergosterol profile. Of the molecules tested, citral was one of the most effective at its MIC causing a 99% reduction in the total ergosterol content. One may attribute the divergent results to the methodological differences, since in the cited work the effect on sterol profile was evaluated by sterol quantitation method.

In a recent study Tao et al. [22] showed that citral considerably impaired ergosterol biosynthesis in cells of* Penicillium italicum*, significantly decreasing lipid levels, suggesting that the plasma membrane may well be an important citral antifungal target.

More recently Zhou et al. [21] evaluated the antifungal activity of three volatile compounds: citral, octanal, and α-terpineol against* Geotrichum citri-aurantii*. It was found that citral in the study was able to significantly inhibit mycelial growth. Antifungal activity was attributed to cell membrane disruption and to consequent loss of cellular components. Another study also showed that citral at a concentration of 200 *μ*g/mL irreversibly damaged cell organelles and the cell membrane of* Trichophyton mentagrophytes* [66].

It is commonly recognized that the presence of sterols in the fungal membrane is essential for the biological activity of amphotericin B, that is, for the formation of transmembrane ion channels. The selective toxicity of the drug for the fungal cell is ascribed to the fact that it is more potent against fungal cell membranes containing ergosterol than against mammalian membranes with cholesterol [67].

## 4. Conclusions

Based on these results, the present study demonstrated that citral has significant antifungal activity against* C. albicans* and revealed that the product concentrations inhibiting growth are the same as those causing death. We highlight that the death kinetics of this compound rise quickly and with increased drug concentrations. Another important aspect was that citral was able to alter the morphology of this species, and the probable mechanism of action does not involve either the cell wall or ergosterol. Therefore, the test product is presented as a relevant and promising antifungal which can be considered as an alternative prototype for production of a new and future antifungal, and thus contributing to the existing arsenal of products with proven antifungal activity against* C. albicans*. Investigations of this nature are important since they provide clearer expectations for future pharmacological studies, with the view of better understanding of citral's mode of action, its toxicity, and its possible therapeutic application.

## Figures and Tables

**Table 1:** MIC values (*μ*g/mL) of drugs in the absence and presence of sorbitol (0.8 M) and ergosterol (400 *μ*g/mL) against *C. albicans* ATCC 76485 and LM-70.

Drugs	Sorbitol		Ergosterol	
	Absence	Presence	Absence	Presence
Citral	64	64	64	64
Amphotericin B^a^	—	—	2	128

^a^Positive control. —: not tested.
